# Translation, adaptation and validation of the Bulgarian version of the King’s Parkinson’s Disease Pain Scale

**DOI:** 10.1186/s12883-021-02392-5

**Published:** 2021-09-15

**Authors:** Galina Stoyanova-Piroth, Ivan Milanov, Katerina Stambolieva

**Affiliations:** 1 St. Naum Hospital of Neurology and Psychiatry, Medical University, 1, Louben Roussev str., 1113 Sofia, Bulgaria; 2 Institute of Neurobiology, Bulgarian Academy of Sciences, Sofia, Bulgaria

**Keywords:** Parkinson’s Disease, Pain, KPPS, Cross-cultural adaptation

## Abstract

**Background:**

The purpose of the present study was to translate and cross-culturally adapt the King’s Parkinson’s Disease Pain Scale (KPPS) into Bulgarian and to investigate its psychometric properties in order to provide a validated Parkinson’s disease-specific pain instrument in Bulgarian language (KPPS-BG).

**Methods:**

Translation into Bulgarian and a cultural adaptation were performed to obtain KPPS-BG. A total of 162 patients with idiopathic Parkinson’s disease were screened for pain using the complementary to the KPPS questionnaire – King’s Parkinson’s Disease Pain Questionnaire (KPPQ). KPPS-BG domain and total scores were calculated and internal consistency, construct validity and test-retest reliability were examined for 129 patients having one or more positive items in the KPPQ-BG.

**Results:**

79.6 % of the patients reported one or more types of pain. The most common type was musculoskeletal pain (83.7 %), followed by nocturnal pain (55.0 %), fluctuation-related pain (50.1 %), radicular pain (43.4 %), chronic pain (31.0 %), discoloration, edema/swelling (27.1 %) and, oro-facial pain (14.3 %). Mean KPPS-BG total score was 21.1 ± 17.3 SD. KPPS-BG showed a good reliability (Cronbach’s alpha 0.75). The test-retest reliability of the KPPS-BG was high and the intraclass correlation coefficient was 0.92, demonstrating а good repeatability. KPPS-BG total score was higher in patients with postural instability gait difficulty motor subtype, compared to tremor-dominant or indeterminate subtype. Significant positive correlations were found between KPPS-BG total score and modified H&Y, Movement Disorders Society Unified Parkinson’s Disease Rating Scale part III.

**Conclusions:**

The KPPS-BG constitutes a reliable, comprehensive and useful tool for pain assessment in native Bulgarian patients with Parkinson’s disease.

## Background

Pain has been reported as symptom of Parkinson’s disease (PD) since the first description of the disorder [1]. In recent years pain is increasingly recognized as important feature of the disease [2] and a cause of reduced quality of life (QoL) [3, 4]. But despite being one of the most troublesome non-motor symptoms (NMS) in patients with both early and advanced PD [5] and, the most frequently reported presenting NMS [6], pain is still under-diagnosed and under-treated [7].

Based on the assessment using general, non-PD specific tools, pain is estimated to occurs in up to 85 % of the patients with PD [7]. This makes pain in PD patients 2–3 times more frequent compared to age-matched healthy individuals [8]. Pains in PD were classified as related or unrelated to the disease [9]. Based on aetiology, Ford [10] classifies pain in PD into five categories: musculoskeletal pain, neuropathic radicular pain, dystonia-related pain, akathisia and primary central parkinsonian pain. Pain can be also categorised as nociceptive, neuropathic and miscellaneous [11].

Until recently, there was no specific validated diagnostic tool for detecting pain in PD. Different specific and non-specific pain scales were used that have not been adopted to and validated for PD patients [9, 12, 13]. This leads to a methodological deficiency for consensual interpretation of the results.

Chaudhuri et al. [14] have created and validated the first specific PD pain scale, named “King’s Parkinson’s disease Pain Scale” – KPPS. Its seven domains include 14 items. KPPS has already been used as a reliable tool available in English language to assess various types of PD-pain [4, 15–17] and as a secondary outcome in two international multicenter, randomized, controlled trials [18, 19]. More recently, the effect of other therapeutic strategies on pain in PD was also assessed by KPPS [20]. Available is an officially translated version in German [21]. Recently, a validated version in Turkish, Hindi and Persian were published [22–24].

The clinical assessment of pain in PD patients in Bulgaria is usually underperformed. Questionnaires or studies on the reliability and validity of instruments assessing pain in patients with Parkinson`s disease in the Bulgarian language do not exist. The aim of the present study is translation, adaptation and validation of the King’s Parkinson’s Disease Pain Scale in Bulgarian language (KPPS-BG) in order to use it as a reliable instrument intended for clinical monitoring the pain in Bulgarian patients with PD.

## Methods

Participants were recruited from the Department of Movement Disorders of the University Hospital for Active Treatment in Neurology and Psychiatry, “St Naum” Sofia, Bulgaria. All inpatient and outpatient treated during the study period were screened for eligibility. Assessment was done by a senior neurologist, specialized in movement disorders.

In the study were enrolled 162 patients aged 18 years or more with diagnosed idiopathic PD based on the UK Parkinson’s Disease Society Brain Bank criteria [25]. All subjects were tested for PD-related pain. The exclusion criteria were: (1) patients with atypical or secondary Parkinsonism; (2) patients with comorbidities causing pain, such as severe osteoarthritis, neuropathy, malignancy, rheumatic diseases or other chronic pain conditions; (3) Mini-Mental State Examination (MMSE) score < 24 [26], indicating significant cognitive impairment, that potentially interferes with the ability to understand the content of the questions.

Demographic and clinical data were collected upon the first visit. All patients were assessed by using the modified Hoehn and Yahr (H&Y) scale for rating the stage of a patient’s disease progression [27] and the Movement Disorders Society Unified Parkinson’s Disease Rating Scale (MDS-UPDRS) part III (motor examination) [28] in on state. The motor subtype was determined based on particular items of the MDS-UPDRS. [29]. Levodopa equivalent daily dose (LEDD) was calculated for each patient according to Tomlinson et al. [30].

All patients gave written informed consents prior to participation, approved by the Ethics Committee of the University Hospital for Active Treatment in Neurology and Psychiatry “St Naum”, Sofia. The study was conducted in accordance with the ethical standards set forth on the Declaration of Helsinki. The participants were free to withdraw from the study at any time.

Following a permission granted by the authors and according to their requirements the original English versions of the KPPS (along with it the proposed King’s Parkinson’s Disease Pain Questionnaire-KPPQ) [31] were translated into Bulgarian language independently by two bilingual persons with medical education. After discussion held among the translators some items of the Bulgarian version of KPPQ and KPPS (KPPQ-BG and KPPS-BG) were edited according to the rules of the Bulgarian language for semantic and grammatical accuracy. By means of a consensus a prefinal version of KPPQ-BG and KPPS-BG were obtained. Two licensed translators unaware of the original version of the KPPQ and KPPS performed independently back translation from Bulgarian to English of KPPQ-BG and KPPS-BG. No significant differences between the translation and retro-translation were found.

Ten patients with various age and educational levels were tested with the pre-final questionnaire and scale (KPPQ-BG and KPPS-BG). They were invited to verbally evaluate the clarity of the content of the items and their comprehensibility. Patients have been filling KPPQ-BG in the presence of a medical specialist, who was helping them out where needed. After filling the questionnaire the patients give answers concerning the severity and frequency of the available pain. The medical specialist calculates the score of each domain and the total score of KPPS-BG.

The following language adaptations were performed: “around their joints” was replaced by “around the joints”, “around the liver, stomach or bowels” was replaced by “in the area of the liver, stomach or bowels” and “aching pain” was replaced by “pain”.

Patients declared that they have not encountered difficulties in completing the questionnaire with the help from the relevant specialist, granted upon such need a request and in answering the questions on the rater-interview–based scale.

The back-translated version of the questionnaire and scale were discussed with the authors of the KPPQ and KPPS. After some amendments the final versions KPPQ-BG and KPPS-BG were again back-translated and approved by the authors for validation and for usе in Bulgarian PD patients.

The KPPS-BG consist of the same 14 items as KPPQ-BG, which are distributed into seven domains as follow: (1) Musculoskeletal pain (1 item); (2) Chronic pain (2 items); (3) Fluctuation-related pain (3 items); (4) Nocturnal pain (2 items); (5) Oro-facial pain (3 items); (6) Discoloration, oedema/swelling (2 items); (7) Radicular pain (1 item). Patients need to rank the described sense of pain for each item by the severity in a 4-point Likert scale (0, none to 3, severe) and frequency in a 5-point Likert scale (0, never to 4, very frequently). The range of possible KPPS-BG total scores is from 0 to 168. Higher score values indicate higher levels of pain.

In total 162 patients with PD were tested with KPPQ-BG. Face-to-face clinical interview was conducted comprising two steps. In the first one, all patients had to fill the KPPQ-BG, answering “Yes” or “No” for all 14 items. Only patients with 1 or more positive answers were evaluated by KPPS-BG. The KPPS-BG domain and total scores were calculated for 129 patients. 43 randomly selected patients were interviewed again after two weeks to evaluate test-retest reliability [32].

### Statistical analysis

Descriptive statistics were used for calculating the mean scores of the KPPS-BG and sociodemographic and clinical data. Item discrimination was evaluated using item-to-total score correlation that is criteria for discarding items. The item-to-total score correlation of 0.20 has been given as the cut-off point below which items can be discarded [33]. Тherefore, only the items that met the correlation greater than 0.20 were retained. The internal consistency of KPPS-BG was determined by estimating Cronbach’s α coefficient and item-total correlation (Spearman’s rank correlation < 0.25 was considered to be weak, while values equal to or greater than 0.76 were considered to indicate a strong relationship). In general, Internal consistency refers to the extent to which all of the items within a scale measure the different aspects of the same attribute. Cronbach’s alpha is often used in assessing the reliability, where Cronbach’s α > 0.7 (Nunnally’s criterion) is considered as sufficiently reliable [34].

The test-retest reliability was evaluated by calculating the intraclass correlation coefficient (ICC) using the two-way mixed effect models according to classification of Shrout and Fleiss (1979) [35]. The reliability was evaluated by classification proposed by Slick (2006) as following: Very high ICCs ≥ 0.90, high ICCs = 0.80–0.89, adequate ICCs = 0.70–0.79, marginal ICCs = 0.60–0.69 and low ICCs < 0.60 [36]. The convergent construct validity was tested with estimation of the correlation of the KPPS-BG total score with other instruments that express PD stage and severity as modified H&Y stage and MDS-UPDRS III. The discriminant validity was established by evaluation of a difference between the KPPS-BG scores and clinical parameters, using Kruskal-Wallis ANOVA.

The analyses were performed using the computer software Statistica 8.0 for Windows (Stat Soft Inc. USA).

## Results

Mean age of participants in the investigation was 65.4 ± 8.4 (range 40–80 years). There were only few participants younger than 50 years (4.7 %). The majority of the PD patients were within the age range of 50–70 years (63.5 %). Both sexes were equally represented. The mean duration of the disease was 6.6 ± 5.7 years. Sixty-three patients (48.8 %) reported disease durations of 6–10 years and 22 (17 %) of over 10 years. Twenty-three patients (17,8 %) were newly diagnosed and drug naive. Sociodemographic and clinical characteristic of patients with KPPS-BG score > 0 are presented in Table 1.

**Table 1:** Sociodemographic and clinical characteristic of patients with PD and pain (*n*=129)

**Variable**	**Values**
Sex, n (%)	
Male	65 (50.4)
Female	64 (49.6)
Age, years (mean ± SD)	65.4 ± 8.4
Education, n (%)	
primary school	6 (4.7)
secondary school	88 (68.2)
university	35 (27.1)
Marital status, n (%)	
single	18 (14.0)
married	84 (65.1)
widowed	27 (20.9)
Duration of disease, years (mean ± SD)	6.6 ± 5.7
Mean age of PD onset (years)	58.7 (10.2)
Motor subtype, n (%)	
Tremor-dominant (TD)	59 (45.7)
Postural Instability Gait Disorder (PIGD)	65 (50.4)
Indeterminate	5 (3.9)
Modified H&Y stage, median (range)	2.0 (1-4)
MDS-UPDRS III	31.4 ± 11.1
LEDD, mg (mean ± SD)	621.9 ± 500.1
Range	(0-1775 mg)

*SD* standard deviation; *PD* Parkinson’s Disease; *MDS-UPDRS III* Movement Disorders Society Unified Parkinson’s Disease Rating Scale part III (motor examination); *LEDD* Levodopa Equivalent Daily Dose

All 129 patients with symptoms of pain over the last month completed KPPQ-BG manually. Based on the patients’ answers, pain severity and frequency were quantitatively measured and the КPPS-BG score was calculated. Missing information on any of the items was not observed (analysis of missing data not supplied). Eight of the patients (6.2 %) had ambiguities related to the words “abnormal” and “involuntary” in question number 5 of the KPPQ-BG and additionally explanations were provided by the interviewing medical specialist. In some cases, additional explanations were required for other items.

There were no significant differences between the distribution of the positive responses of the Bulgarian and mixed British and Romanian populations using English version of KPPS, for most of the questions. Only in item 4 and domain 4 the responses of the Bulgarian patients were significantly different (Table 2).

**Table 2:** Proportion of patients with positive responses of KPPS-BG (n = 129) and comparison with the original KPPS (*n* = 300) [31]

Items	KPPS-BG	KPPS English	Fisher exact test
1. Pain around joints (musculoskeletal)	83.7	81.3	0.46
2. Pain related to internal organ	20.2	21.0	0.81
3. Generalised non-specific pain in the stomach area	13.9	17.7	0.11
4. Pain deep within the body	12.4	31.7	**0.001**
5. Dyskinetic pain	16.3	17.0	0.86
6. Painful muscle cramps in a specific area during “off” period	41.9	32.7	0.08
7. Generalized “off” period pain	17.8	22.3	0.29
8. PLM or RLS-associated pain	24.8	28.0	0.49
9. Pain while turning in bed at night	47.3	47.7	0.94
10. Pain when chewing	4.7	6.7	0.43
11. Pain due to grinding teeth during the night	5.1	5.7	0.25
12. Burning sensation in the mouth	3.3	2.7	0.31
13. Burning pain in the limbs	18.6	18.3	0.93
14. Shooting pain/pins & needles	43.4	41.7	0.74
**Domains**			
1. Musculoskeletal pain	83.7	81.3	0.46
2. Chronic pain	31.0	40.0	0.08
3. Fluctuation-related pain	50.1	44.0	0.37
4. Nocturnal pain	55.0	66.0	**0.03**
5. Oro-facial pain	14.3	13.3	0.78
6. Discoloration, edema/swelling	27.1	30.3	0.69
7. Radicular pain	43.4	41.7	0.74

*KPPS-BG* Bulgarian version of the King’s Parkinson’s Disease Pain Scale; *KPPS* King’s Parkinson’s Disease Pain Scale; *PLM* periodic limb movements; *RLS* restless legs syndrome

Internal consistency was calculated using Cronbach’s alpha coefficient, based on the data obtained from the first examination (n = 129). The value of Cronbach’s alpha for the total scale KPPS-BG was 0.75, which showed good internal reliability. The item-total correlations were 0.21 or greater for 13 items, ranged from 0.21 (item 3) to 0.72 (item 14). The item 12: “Burning sensation in the mouth” have values of item-total correlations < 0.20, which defines it as poorly informative and gives reason to exclude it from the subsequent analysis (Table 3).

**Table 3:** Internal consistency reliability and test-retest reliability (Intraclass correlation coefficients and 95 % confidence intervals) of KPPS-BG

Items of KPPS-BG	Item-total correlations (IT-TC) (n = 129)	Cronbach’s α if item deleted	ICCs (CI) (n = 43)elet
1. Pain around joints (musculoskeletal)	0.46	0.75	0.84 (0.67–0.90)
2. Pain related to internal organ	0.31	0.72	0.78 (0.62–0.88)
3. Generalised non-specific pain in the stomach area	0.21	0.74	0.84 (0.66–0.89)
4. Pain deep within the body	0.38	0.75	0.97 (0.92–0.99)
5. Dyskinetic pain	0.68	0.73	1 (1–1)
6. Painful muscle cramps in a specific area during “off” periods	0.41	0.73	0.94 (0.85–0.91)
7. Generalized “off” period pain	0.49	0.79	0.85 (0.72–0.91)
8. PLM or RLS-associated pain	0.52	0.77	0.97 (0.87–0.99)
9. Pain while turning in bed	0.71	0.73	0.95 (0.91–0.98)
10. Pain when chewing	0.64	0.71	1 (1–1)
11. Pain due to grinding teeth	0.46	0.75	0.98 (0.96–0.99)
12. Burning sensation in the mouth	0.15	0.74	0.80 (0.61–0.87)
13. Burning pain in the limbs	0.66	0.73	0.98 (0.93–0.99)
14. Shooting pain/pins & needles	0.72	0.71	0.97 (0.91–0.99)

*KPPS-BG* Bulgarian version of the King’s Parkinson’s Disease Pain Scale; *PLM* periodic limb movements; *RLS* restless legs syndrome

The test-retest reliability of the KPPS-BG was high and the intraclass correlation coefficient (ICC) was 0.92 (confidence interval (CI) = 0.82–0.98). All items showed high positive correlations between the first and second exam (range from 0.78 to 0.98), which exceeded the recommended value of 0.75 (Nunnally) and show that items contained in the KPPS-BG had a good repeatability (Table 3).

Convergent validity of the domains and total scores of the KPPS-BG with other instruments that assess disability status of PD patients was estimated using Spearman’s rank correlation coefficients (r_s_) (Table 4). The mean scores of domains and total score of the KPPS-BG are presented in Table 4.

**Table.4:** Domain scores and total score of the KPPS-BG and correlations with other PD specific scales

KPPS-BG scores	Mean score (SD)	Median	Range	Modified H&Y	MDS-UPDRS III
1. Musculoskeletal pain	5.13 (3.59)	6	0–12	0.24*	0.26*
2. Chronic pain	1.57 (3.13)	0	0–18	0.05	0.03
3. Fluctuation-related pain	4.46 (6.51)	1	0–36	0.61*	0.54*
4. Nocturnal pain	5.35 (6.33)	0	0–24	0.27*	0.2
5. Oro-facial pain	0.88 (3.03)	0	0–12	0.31*	0.24*
6. Discoloration, edema/swelling	1.41 (2.72)	0	0–12	0.13	0.11
7. Radicular pain	2.17 (3.28)	0	0–12	0.13	0.04
**Total score**	21.11 (17.32)	17	1–90	0.47*	0.42*

* Statistical significance of Spearman correlation coefficient, *p* < 0.05; Modified H&Y, modified Hoehn and Yahr scale; *MDS-UPDRS III* Movement Disorders Society Unified Parkinson’s Disease Rating Scale part III (motor examination); *KPPS-BG* Bulgarian version of the King’s Parkinson’s Disease Pain Scale; *SD* standard deviation

**Fig. 1:**
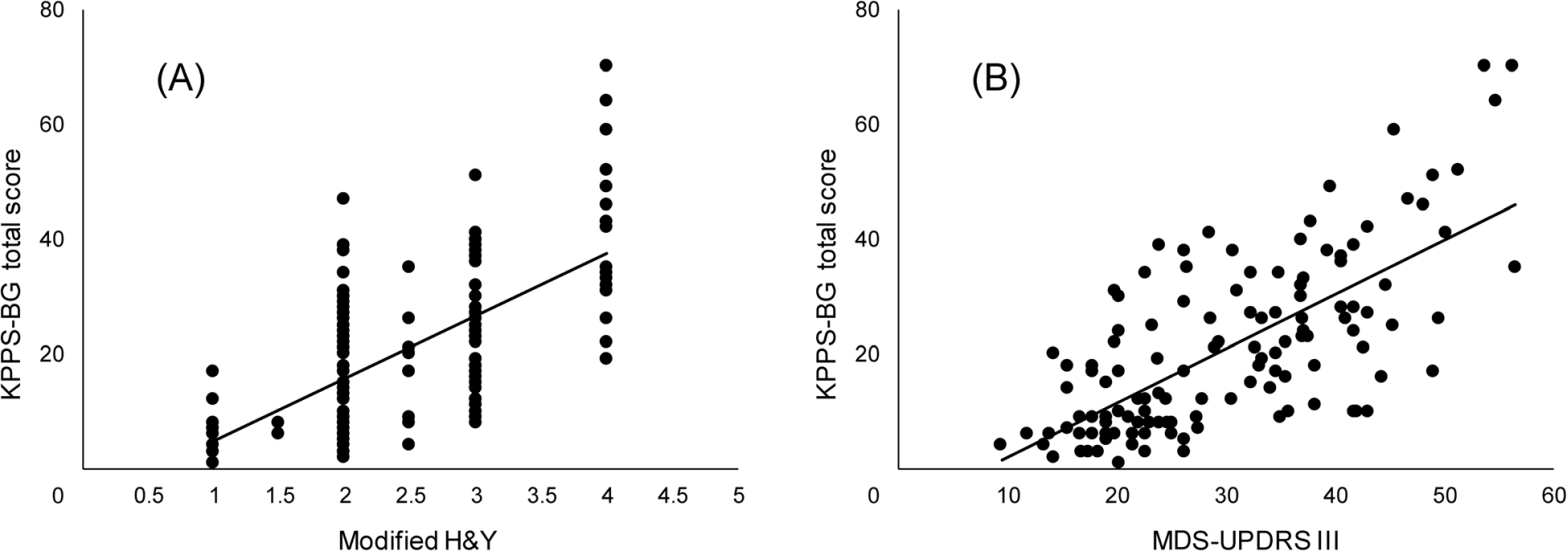
Scatterplot showing the correlations between KPPS-BG Score and modified H&Y (**A**) and MDS-UPDRS III (**B**). The individual points represent individual cases. KPPS-BG, Bulgarian version of the King’s Parkinson’s Disease Pain Scale; Modified H&Y, modified Hoehn and Yahr scale; MDS-UPDRS III, Movement Disorders Society Unified Parkinson’s Disease Rating Scale part III (motor examination)

Figure 1 shows a scatterplot with the positive correlation between KPPS-BG Score and modified H&Y (A) and MDS-UPDRS III (B).

Discriminate construct validity was tested by difference between KPPS-BG total score and motor subtype of PD using Kruskal-Wallis ANOVA with three levels (Tremor-dominant (TD), Postural Instability Gait Disorder (PIGD) and Indeterminate). There was a significant effect of the PD motor subtype on the KPPS-BG total score (H (2, 129) = 26.26, *p* < 0.001). A post hoc Mann-Whitney U-test showed significant difference of KPPS-BG total scores between patients with TD subtype vs. PIGD (*p* < 0.001) only. The mean score of TD was 13.5 ± 2.5, median 7, for PIGD was 28.3 ± 19.1, median 23 and Indeterminate subtype- 15.4 ± 7.1, median 16. That result shows a good discriminative validity of KPPS-BG.

## Discussion

Pain is a subjective, physiological and psychological experience, influenced by sociocultural factors [37]. In the Parkinson population, pain is an important and frequently reported NMS [10]. In order to investigate the types of pain and its frequency in PD for the Bulgarian patients, we translated and validated the only one PD-specific scale for pain- KPPS. The results of the present study demonstrate that KPPS-BG had a good reliability and validity. Cronbach’s α of total scale was 0.75, which indicates a good level of internal consistency similar to those reported in the original publication (0.78) [14], by Rodríguez-Violante et al. (0.74) [15], and by Soyuer et al. (0.827) [22]. Taghizadeh et al. found Cronbach’s 0.88 [24].

Almost 80 % of our patients reported one or more types of pain. In a recently published cross-sectional study, the authors reported a pain frequency of 88.6 % [15]. Silverdale et al. [16] also using KPPS in patients with early/moderate PD found 85 % of participants to experience pain. Slightly lower frequency was revealed in Brazilian cohort (70.3 %) [17]. One German study based on a self-developed Parkinson’s Disease Pain Questionnaire revealed up to 95 % prevalence of pain in PD patients [38]. Lower prevalence is reported in Indian cohort (52.1 %) [23] although mean disease duration and mean H&Y are similar to ours.

In the present study, the most common type of pain was musculoskeletal (83.7 %), which is in line with the results of some previous studies [17, 23, 31], but higher than others [15]. Independent of the use of a PD specific pain assessment tool, this is the most common type of pain described in the literature (frequency range: 40–90 %) [7, 11].

Significant differences between the Bulgarian and other populations were observed for item 4 and nocturnal pain domain. Pain deep within the body (item 4) refers to the central pain. It has been reported by 12 % of our patients. These results are in line with other studies where this type of pain was estimated to be present in up to 10 % of PD patients [11]. However, they are significantly lower than the results retrieved applying KPPS [23, 31]. Possibly, these differences arise from patient population and socio-cultural peculiarities.

55.0 % of patients in the present study suffered from nocturnal pain. This domain shows a large variability among different studies [15, 31] The third most common type of pain was fluctuation-related pain (50.1 %), followed by radicular pain (43.4 %) and chronic pain (31.0 %). 27.1 and 14.3 % of the patients, respectively, reported discoloration edema/swelling pain and orofacial pain. Likewise, these two pain categories were least common in other studies [15, 23, 31].

The mean scores of all domains of the KPPS-BG were similar to those observed in the original validation [14] except for chronic pain (domain 2) where the patients’ score of the original KPPS was significantly higher (3.37 ± 5.53) in comparison with the score of the Bulgarian population.

The mean total score of KPPS-BG was 21.1 ± 17.3 and median 17, which is within the range of those reported by other authors [14, 15]. In the recently published Indian validation, the mean total KPPS score was found to be 16.02 ± 10.57 [23].

Item 12. Burning sensation in the mouth demonstrated an item-to-total score correlation below 0.20. Hence, this item was determined as low informative and unreliable for the Bulgarian population.

We have observed a statistical correlation between modified H&Y stage, MDS-UPDRS III and the total score KPPS-BG (Tabale 4). Тhe observed positive correlations reflect the domination of the musculosceletal, fluctuation-related, nocturnal and orofacial pain in the overall score. Similar data was published [14, 15, 23]. Contrariwise, Silverdale et al. [16] found no correlation between UPDRS III and KPPS most domains. This is probably due to different clinical characteristics: shorter mean disease duration, and UPDRS IV indicated only a low number of PD patients suffering from fluctuations. De Mattos et al. [17] analysed only 38 patients also with shorter mean disease duration and lower UPDRS III score and reported no significant correlation of UPDRS III and KPPS total score.

The significant difference of KPPS-BG total scores between patients with various motor subtypes of the disease is published also for the Mexican population [15]. Patients with TD subtype were scored lowest.

KPPS-BG detected pain as an initial symptom of the disease in 5.43 % of the patients.

6.2 % of the participants required further clarification with regard to the words “abnormal” and “involuntary” in item 5. In some cases, additional explanations were required for other items. This allows us to propose the use of the questionnaire and scale in the presence of a medical specialist who is trained to recognize the specific taxonomy of pain in PD.

Due to the lack of consensus on the diagnosis and therapy, pain in PD is still insufficiently managed in routine clinical practice. In the present study, this is reflected by means of the finding that 79.63 % of the participants reported one or more types of pain, but only 10.85 % received medications for pain relief.

The current study has some limitations: The main one is the small sample size for the test-retest reliability. Only those patients that came to a follow-up visit two weeks after the first assessment and could be examined by the same senior neurologist were included. This could be a potential source of bias. However, the results are in line with previous studies. Second, the number of patients with advanced disease (modified H&Y stage 4 and 5) is underrepresented, mainly due to the exclusion criteria. Third, the methodological design of the study is not а case-control. Therefore, no control group was included. All assessments are made in on medication phase.

## Conclusions

Pain is a common, disabling and influencing the patient’s QoL non-motor symptom in PD, but still a challenge for the clinicians. Standardized outcome measurements would help developing consensus on pain management in PD. As many other tools, KPPS was provided in English language and tailored to suit the specifics of the Anglo-Saxon culture. Translation and application in another language requires linguistic and cultural adaptation and a statistical validation. In this study, the KPPS was translated, cross-culturally adapted, and validated into Bulgarian language for the first time. Results of the present study demonstrate that the KPPS-BG has good reliability and validity. The Cronbach’s α 0,75 of total scale indicate good level of internal consistency. Our results showed that KPPS-BG constitutes a useful pain assessment tool evaluating the frequency and severity of pain associated with PD. It can also be useful for monitoring the therapeutic management of PD-related pain. This study supports the use of the KPPS as an intercultural comparable assessment tool.

## Data Availability

The datasets used and analyzed during the current study are available from the corresponding author on reasonable request.

## Abbreviations

KPPS: King’s Parkinson’s Disease Pain Scale KPPS
KPPS-BG: Bulgarian version of the King’s Parkinson’s Disease Pain Scale
KPPQ: King’s Parkinson’s Disease Pain Questionnaire
PD: Parkinson’s disease
QoL: Quality of life
NMS: Non-motor symptom
MMSE: Mini-Mental State Examination
H&Y: Hoehn and Yahr
MDS-UPDRS: Movement Disorders Society Unified Parkinson’s Disease Rating Scale
LEDD: Levodopa equivalent daily dose
ICC: Intraclass correlation coefficient
CI: Confidence interval
TD: Tremor-dominant
PIGD: Postural Instability Gait Disorder
SD: Standard deviation
PLM: Periodic limb movements
RLS: Restless legs syndrome
